# The Final Days of Paracas in Cerro del Gentil, Chincha Valley, Peru

**DOI:** 10.1371/journal.pone.0153465

**Published:** 2016-05-04

**Authors:** Henry Tantaleán, Charles Stanish, Alexis Rodríguez, Kelita Pérez

**Affiliations:** 1 Cotsen Institute of Archaeology, University of California Los Angeles, Los Angeles, California, United States of America; 2 Escuela Superior Politécnica del Litoral, Guayaquil, Ecuador; 3 Santa Fe Institute, Santa Fe, New Mexico, United States of America; 4 Escuela Académico Profesional de Arqueología, Universidad Nacional Mayor de San Marcos, Lima, Peru; 5 Escuela Académico Profesional de Arqueología, Universidad Nacional de Trujillo, La Libertad, Peru; New York State Museum, UNITED STATES

## Abstract

This article describes and analyzes a highly significant archaeological context discovered in a late Paracas (400–200 BCE) sunken patio in the monumental platform mound of Cerro Gentil, located in the Chincha Valley, Peru. This patio area was used for several centuries for ritual activities, including large-scale feasting and other public gatherings. At one point late in this historical sequence people deposited a great deal of objects in what is demonstrably a single historical event. This was quickly followed by a series of minor events stratigraphically immediately above this larger event. This entire ritual process included the consumption of liquids and food, and involved the offering of whole pottery, pottery fragments, botanical remains, bone, lithics, baskets, pyro-engraved gourds, mummies, and other objects. We interpret these events as an “abandonment ceremony” or “termination ritual” during the late Paracas period, one that may have lasted for weeks or even months. The subsequent Topará occupation at the site (ca. 200 BCE- AD 100) involved the architectural enhancement of the mound area, but the pattern of use of the patio itself ended. Such a termination ritual signals a reorganization in the regional political structure of Paracas society.

## Introduction

**Fig 1:**
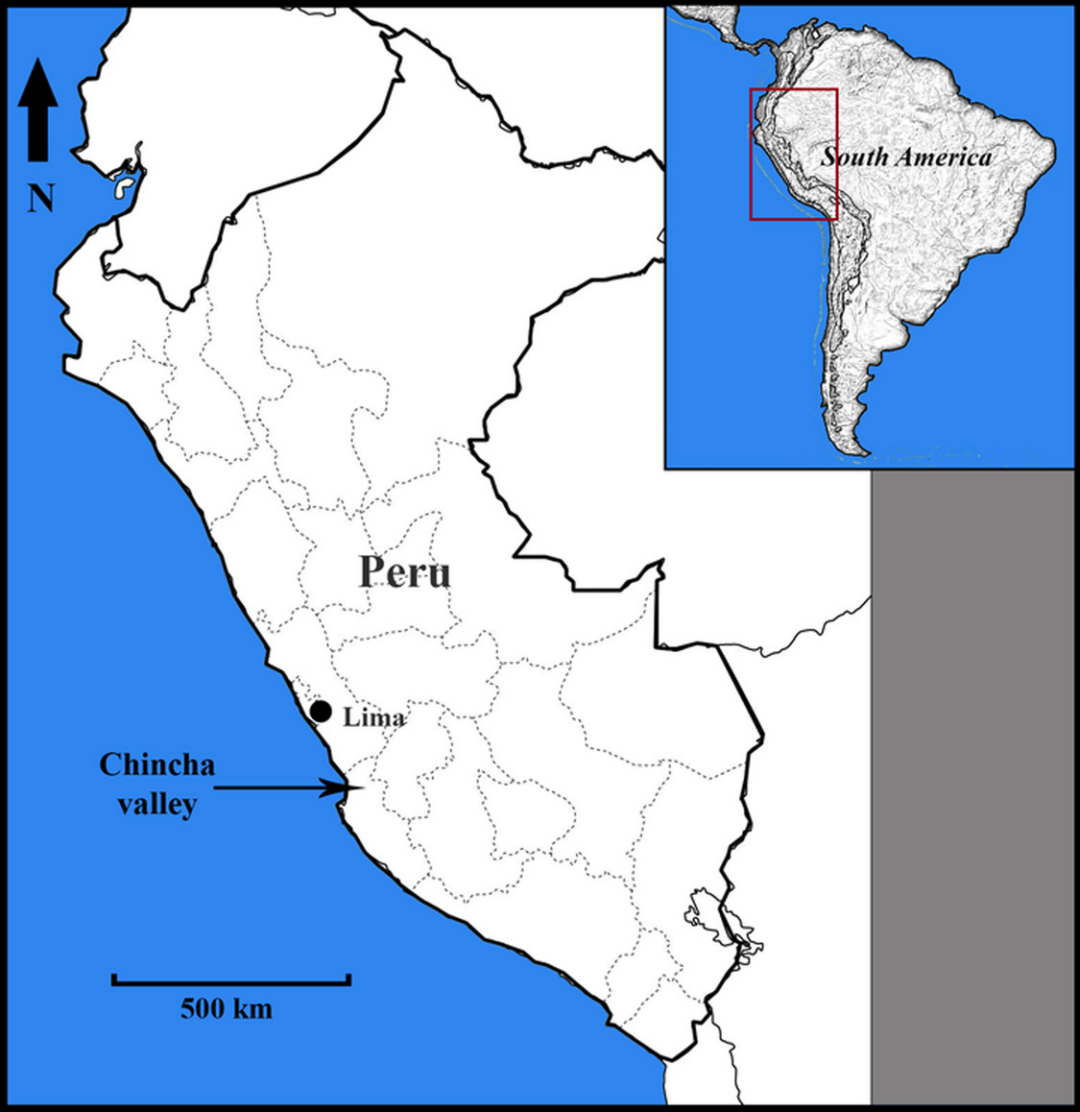
View of South America and Peru. Geographic position of Chincha valley.

Paracas is the oldest archaeological complex society of the southern coast of Peru and is the direct antecedent of the famous Nazca culture located a few hundred kilometers to the south (Fig 1). It is famous for its spectacular pottery, gourd and textile arts. Two generations of research teach us that Paracas developed around 800 BC and continued up to around 200 BC, where it evolved into, or was replaced by the Topará culture that survived for around three more centuries.

**Fig 2:**
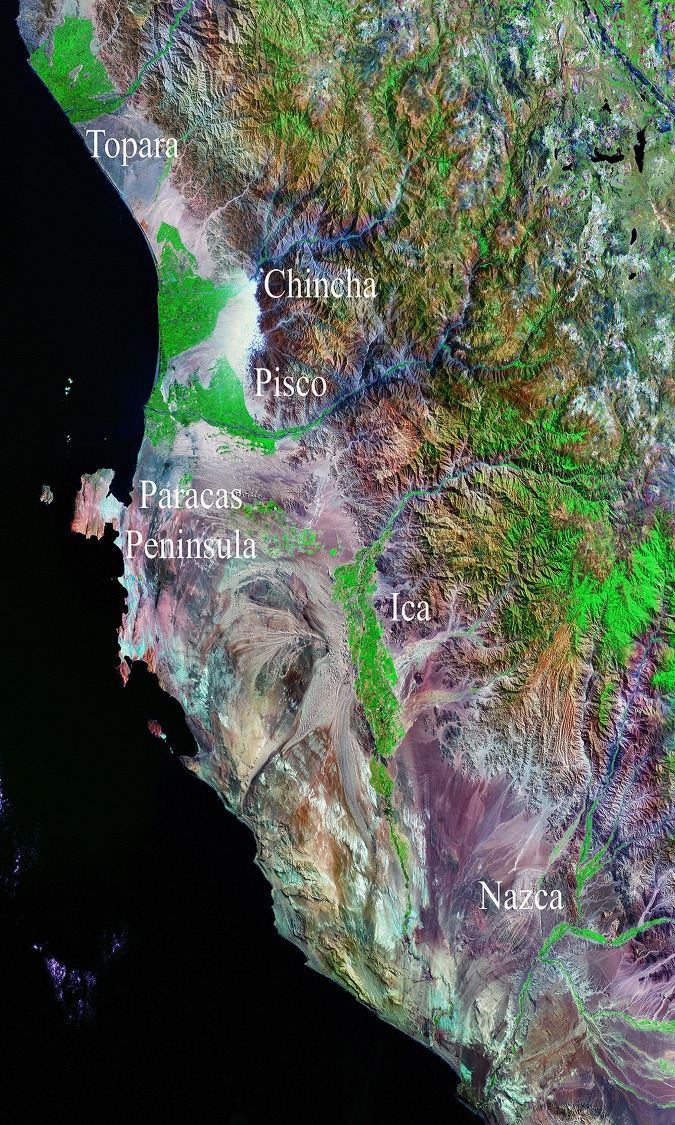
Coastal area related with Paracas style. From North to South: Topará creek, Chincha valley, Pisco Valley, Paracas peninsula, Ica valley and Nazca basin (Lansat image, NASA)

**Fig 3:**
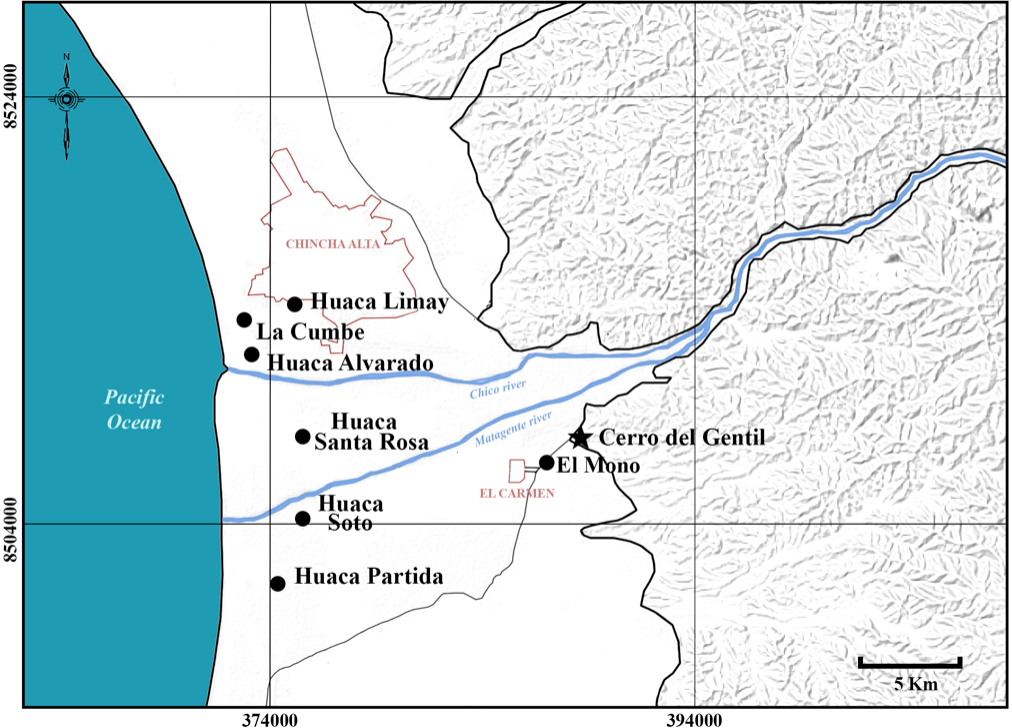
Paracas Sites in Chincha Valley mentioned in the text

Earlier research established the fact that Paracas materials were distributed between the valleys of Cañete and Nazca in the rich valleys along the Peruvian coast (Fig 2). Ernst Middendorf [1], Max Uhle [2], and Alfred Kroeber [3], were pioneers in archaeological studies in Chincha. Uhle and Kroeber made excavations on Paracas sites such as Huaca Alvarado, La Cumbe and Huaca Santa Rosa, all large monuments located in the lower valley (Fig 3). Dwight Wallace surveyed the valley in the middle of the 1950s [4–8]. He recorded many Paracas sites including Cerro del Gentil and assigned it the registry number of PV57-59 [4]. Later, in the 1980s, Luis Lumbreras and members of the Instituto Andino de Estudios Arqueológicos (INDEA), conducted an important research program in the valley. According to Canziani [9, 10], they found a number of monumental Late Paracas sites characterized by stepped platforms with sunken patios made with conical adobe bricks. These sites were found throughout the valley. Both the site of Cerro del Gentil and the cluster of sites known as El Mono (more properly referred to as Chococota) were investigated by Lumbreras and his team [11]. In particular, Elizabeth Isla excavated at Mound C at El Mono and produced a thesis on this important site [12] and other sectors of the Mono sites were tested by the INDEA group.

Most of these earlier investigators argued that Paracas society was centered in the Ica valley and the adjacent Paracas Peninsula [13]. The work of the one of the founders of modern Peruvian archaeology, Julio C. Tello [14, 15], established this view buttressing it with spectacular finds from the Peninsula that included elaborate burials with beautifully executed works of art.

More recent research [9–11] has forced us to change this interpretation. Both Luis Lumbreras [11] and Michael Moseley [16] hinted that the Chincha valley was a major, if not the main center of the Paracas polity. Dwight Wallace [4–6] conducted extensive surveys in Chincha; his data indicated that the Paracas settlement of the valley was quite extensive. This indirectly provided support for Lumbreras’ and Moseley’s ideas about the geographical location of the center of the Paracas polity.

**Fig 4:**
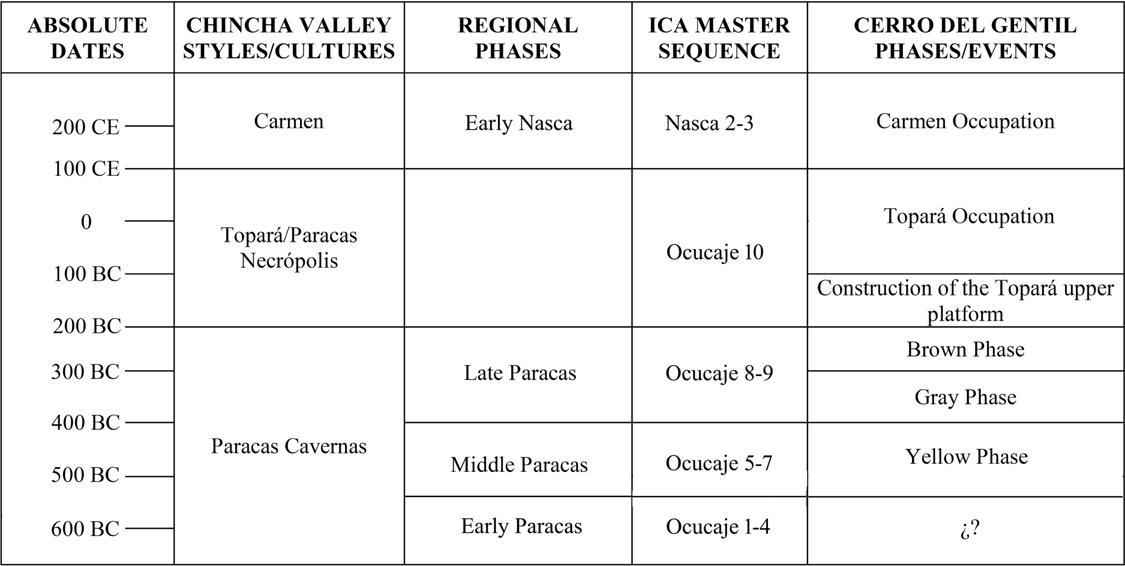
Chronological chart correlating Styles, Regional Phases, Ica Master Sequence and Cerro Gentil phases/events.

It is in this research context that we excavated a sunken patio on Cerro del Gentil. The sunken court was built by Paracas peoples as early as 550 BCE, and quite likely earlier (Fig 4). They deposited pottery, plants of various kinds, shellfish, bone, lithic tools, textiles, humans, and other items in the sunken court. After several centuries of use as a feasting and place of ritual offerings, there was a large and sustained series of events that proved to be the last by Paracas peoples at Cerro del Gentil. This last use of this patio was a type of abandonment ceremony that ended several centuries of intensive ritual use of this place. During this event, this space was filled and the surface area was later reused by people with Topará material culture. In this article, we describe and compare the relationship between the last use of this patio and the earlier ones. We use a theory of feasting to help us understand the strategies of various social and political groups in complex, nonstate contexts such as Paracas.

### Recent research by the Programa Arqueológico Chincha

Our multidisciplinary archaeological team conducted extensive survey, mapping and excavations in the valley effectively building on the work of the INDEA group and the earlier researchers. Our team discovered and mapped an area of about 30 km^2^ of geoglyphs in the pampa above Chincha [17]. We also test excavated three mounds at Chococota and confirmed Isla’s results in at least one of these sites. We established that the entire site cluster was almost completely Paracas in date [17]. We likewise excavated the large courts at the coastal plain site of Huaca Soto [18] and tested modern day agricultural areas between the two main mounds at the site. Huaca Soto was built in the Paracas period, as suggested by Canziani, and the rest of these large huacas in the floodplains near the ocean [9, 10] are most likely Paracas as well.

These new data [14, 17–21] support Lumbreras’ and José Canziani’s observations that there exist a series of massive platform structures built in the Paracas style in Chincha, monuments not found in Ica or other areas to the south. Our survey in the pampa areas above the valley likewise discovered an elaborate geoglyph and mound complex that definitively dates to the later Paracas periods [17]. Thus, the Paracas occupation of the Chincha Valley includes massive platform mound sites in the lower valley, smaller but equally complex platform mounds in the pampa above the valley, a set of geoglyphs, smaller ceremonial sites and an indeterminate number of nonmonumental domestic sites scattered throughout the valley. This recent work therefore strongly supports the earlier suggestions that Chincha was indeed the political center of the Paracas polity, with Ica being a peripheral zone and the Paracas Peninsula possibly the location of elaborate elite burial grounds for people from around the region from Nazca to Chincha.

In short, a new model is emerging about the settlement pattern of the Paracas period in the Chincha valley: people lived and worked fields and fished the sea at the base of the valley where they built very large platform mounds. Test pits around one of these—Huaca Soto—indicate that Paracas people lived and farmed there between the two mounds at the site. They not only built several very large mounds but they also created a ritualized landscape in the pampas above the valley where they constructed much smaller complementary ritual sites, such as Cerro del Gentil.

## Material and Methods: Site and Samples

### Cerro del Gentil

Cerro del Gentil is located on the south east side of the valley (Fig 3). The mound is relatively small compared to other large monumental platform mounds in the lower valley such as Huaca Soto, Huaca Santa Rosa or Huaca Partida. The main building of Cerro del Gentil (Mound A) is about 6300 m^3^ in volume while the site of Huaca Soto, for instance, is about 182,000 m^3^.

**Fig 5:**
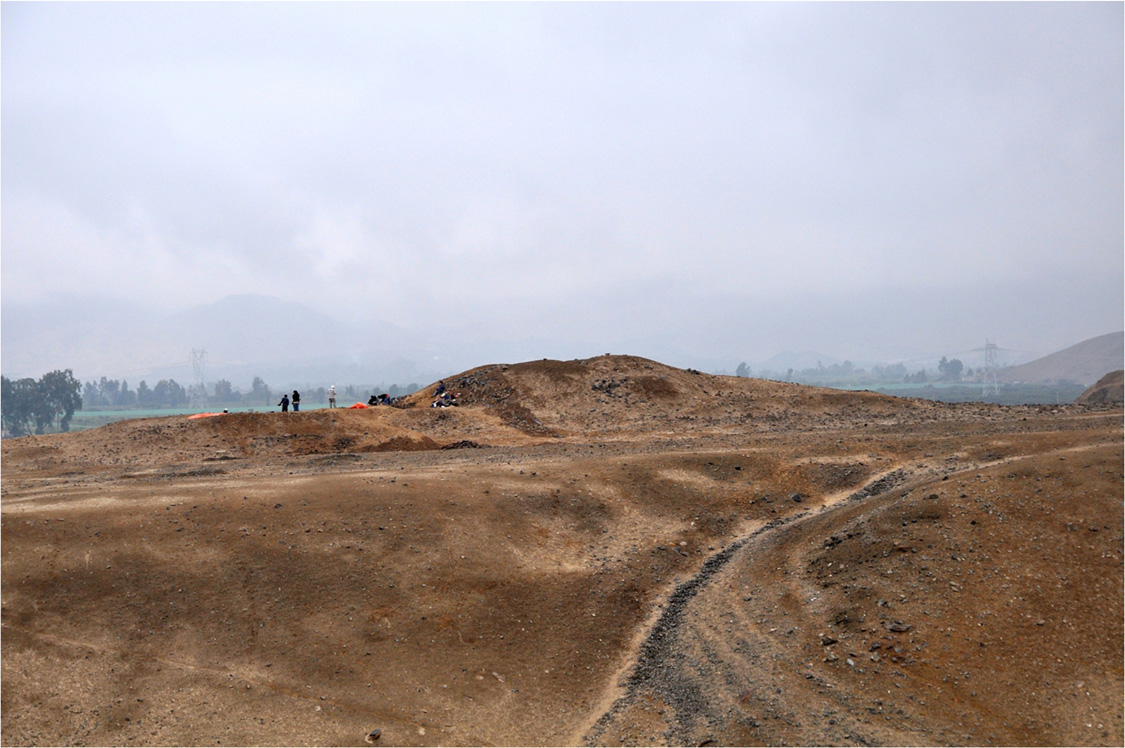
Cerro del Gentil, view from south-west. People in this picture are standing on the sunken court edge.

The site is constructed on the border of a natural terrace above the agricultural lands. It is located at an altitude of approximately 192 m.a.s.l. (Fig 5). The site is composed of two platform mounds, the first approximately 70 x 30 m. and 6 m. in the highest point (Mound A); the other platform is 30 m. x 20 m. and 2 m. high (Mound B).

**Fig 6:**
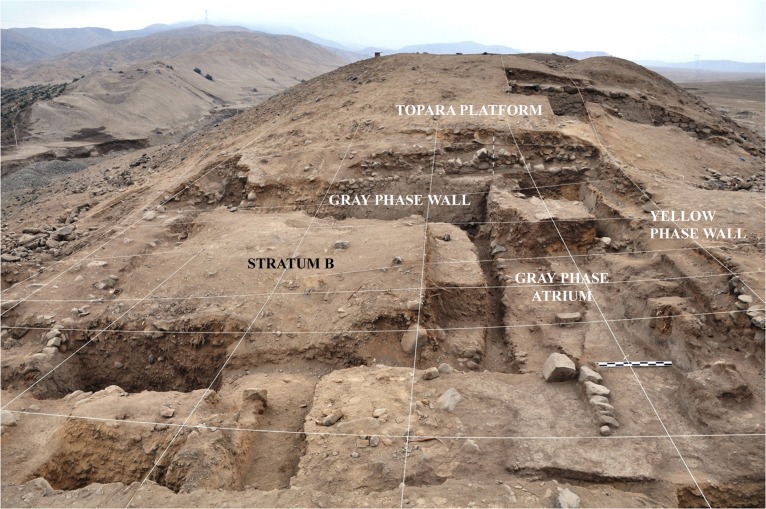
View from the west of Cerro del Gentil sunken patio.

**Fig 7:**
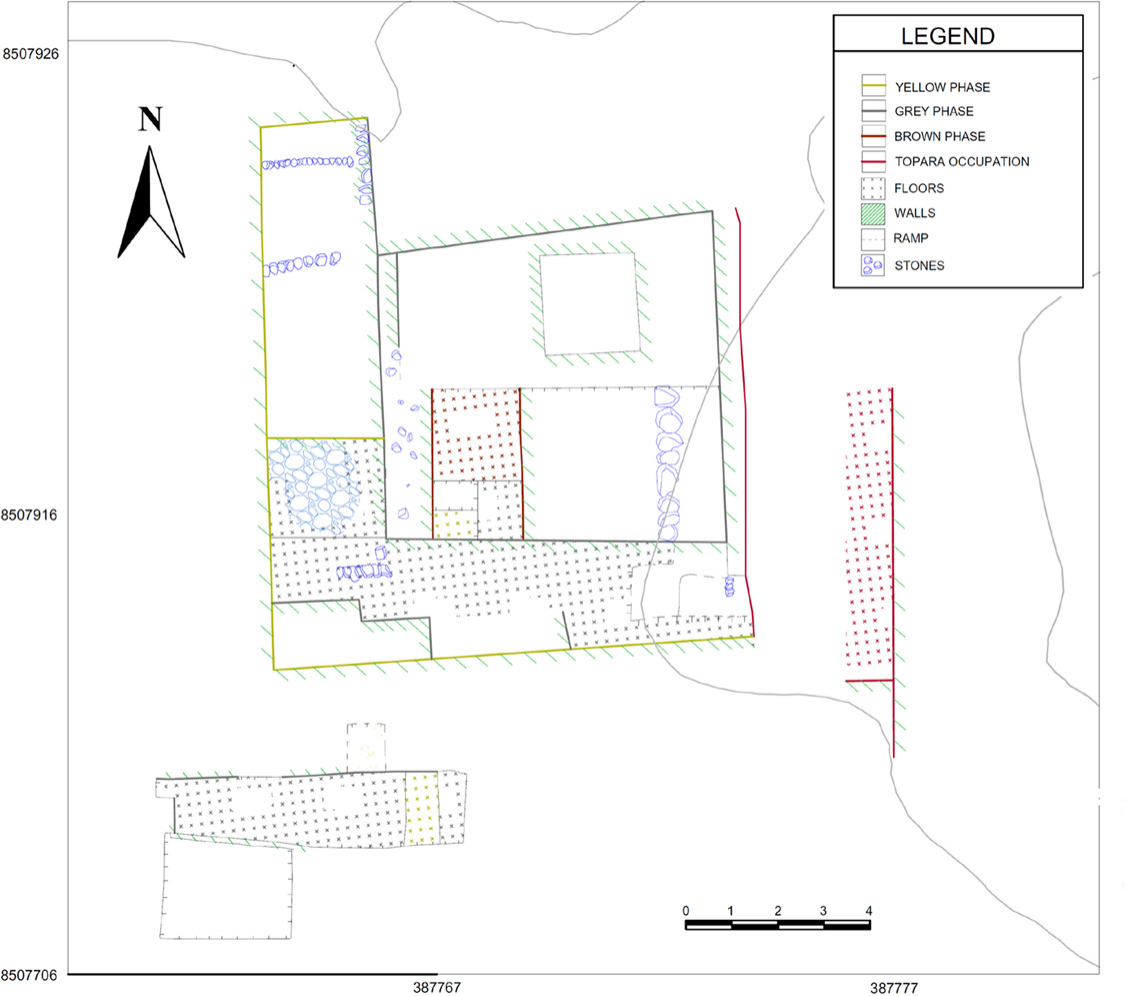
Plan of Sunken Court of Cerro del Gentil based on our excavations.

Cerro del Gentil is one of the smaller monumental Paracas sites in the middle Chincha Valley. In the last few years, our project has conducted intensive excavations on the site. We confirmed that the sunken areas suggested by Canziani to be ritual patios were in fact such constructions. The patio that we excavated on Cerro del Gentil had a number of discrete periods of construction, use, reconstruction and burial [21] (Figs 6 and 7). It was one of the two or three sunken patios on the Cerro del Gentil platform. This architectural complex was an integral component for the Paracas rituals conducted at the site.

**Fig 8:**
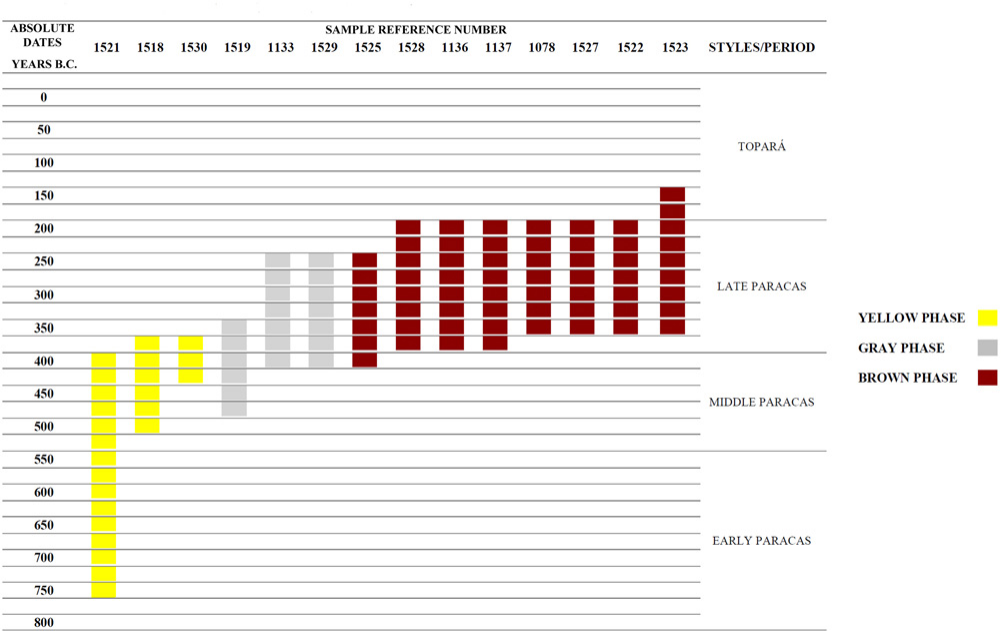
Visual Graphic of Radiocarbon Dates from Cerro del Gentil. Date ranges are in 2 Sigmas.

We intensively excavated in Mound A [19–21] and conducted smaller test units on Mound B. Our excavations indicate that the site was first built in the early to middle Paracas period (Table 1 and Fig 8). This mound was used mainly for ceremonial activities. There is little evidence for a permanent occupation at the site during the Paracas Period. As mentioned, there is a later Topará occupation that remodeled the mound, followed by a squatter Carmen settlement (AD 100–400) off of the mound proper (Fig 4). Late Intermediate Period (AD 1100–1450) and possibly Late Horizon (1450–1532) tombs ring the former monumental areas of the site.

**Table 1:** Radiocarbon Dates from Cerro del Gentil.

Laboratory ID	Reference Number	Locus	Phase	Material	δ^13^C ‰	^14^C (BP)	Calibrated 2-sigma ^a^
UCIAMS-162406	T-1521	Locus 348	Yellow Phase	Botanic	-10	2480±20	750–615 (0.33) cal BCE 590–405 (0.67) cal BCE
UCIAMS-162403	T-1518	Locus 129	Yellow Phase	Maize leaf	-10,6	2395±20	515–380 cal BCE
UCIAMS-162415	T-1530	Locus 149	Yellow Phase	Carbonized wood	-25,4	2360±20	415–360 cal BCE
UCIAMS-162404	T-1519	Locus 298	Gray Phase	Vegetal matter	-11,1	2370±20	475–360 cal BCE
UCIAMS-137882	T-1133	Locus 77	Gray Phase	Textile	^b^	2340±20	410–355 (0.91) cal BCE 285–255 (0.09) cal BCE
UCIAMS-162414	T-1529	Locus 157	Gray Phase	Charcoal	-24,8	2350±20	410–260 cal BCE
UCIAMS-162410	T-1525	Locus 97–2	Brown Phase	Textile	^b^	2330±20	405–355 (0.82) cal BCE 290–240 (0.18) cal BCE
UCIAMS-162413	T-1528	Locus 298	Brown Phase	Vegetal cord	-22,7	2270±20	385–210 cal BCE
UCIAMS-137884	T-1136	Locus 82	Brown Phase	Textile	^b^	2260±20	370–205 cal BCE
UCIAMS-137885	T-1137	Locus 113	Brown Phase	Textile	^b^	2230±20	360–200 cal BCE
UCIAMS-131979	T-1078	Locus 97	Brown Phase	Textile	-25.6	2220±15	360–200 cal BCE
UCIAMS-162412	T-1527	Locus 82	Brown Phase	Matting	-23,7	2265±20	380–210 cal BCE
UCIAMS-162407	T-1522	Locus 150	Brown Phase	Textile	-22.0	2255±20	365–205 cal BCE
UCIAMS-162408	T-1523	Locus 247	Brown Phase	Textile	-22.3	2220±20	360–155 cal BCE

^a^ Calibration of the 14C age for each measurement utilized CALIB 7.0 program protocols employing the SHcal13 (Southern Hemisphere terrestrial) data set (Reimer et al. 2013). Single interval 2σ range calibration values are expressed for intercepts representing ≥0.95 of the relative area under the probability distribution. If relative area is ≥0.1, that value is listed in parenthesis. In cases of multiple intercepts, the 2σ ranges with relative areas under probability distribution of ≥0.05 are noted in parenthesis for intercept separations of ≥20 yrs. Age ranges are rounded to nearest 5 year increment.

^b^ Insufficient yield of CO2 from combustion to obtain gas sample for δ13C measurement.

The sunken patio at Cerro del Gentil that we excavated is located in the center of the Mound A (Fig 6). Our excavations defined four large construction and/or use phases in this court. The first three are related to the Paracas Cavernas style while the fourth is associated with Topará, a style that is related to Paracas Necrópolis [22]. Our carbon dates indicate that the Paracas Cavernas occupations at Cerro del Gentil began no later than 550 BCE (Table 1 and Fig 8), and it is likely that the first occupations are a century or two older because there are earlier levels where we could not obtain sufficient carbon at this time to date.

A detailed description of these data and the methods that we used is found in other publications [18–20] and we will only schematically summarize them here. Briefly, in the first phase, referred to as the “Yellow phase”, the builders leveled the natural surface with a clay floor and constructed the first patio. This first patio was 12.0 x 12.0 m. and was 2.50 m. deep. We believe that it dates to around 500–400 BCE. In the second “Gray phase”, the patio was remodeled and the size was reduced to 7.0 x 7.0 m. and 2.3 m deep. This episode in the use of the patio dates to the 4^th^ century BCE (Figs 6 and 8 and Table 1).

### The “Brown” phase of the sunken patio at Cerro del Gentil

**Fig 9:**
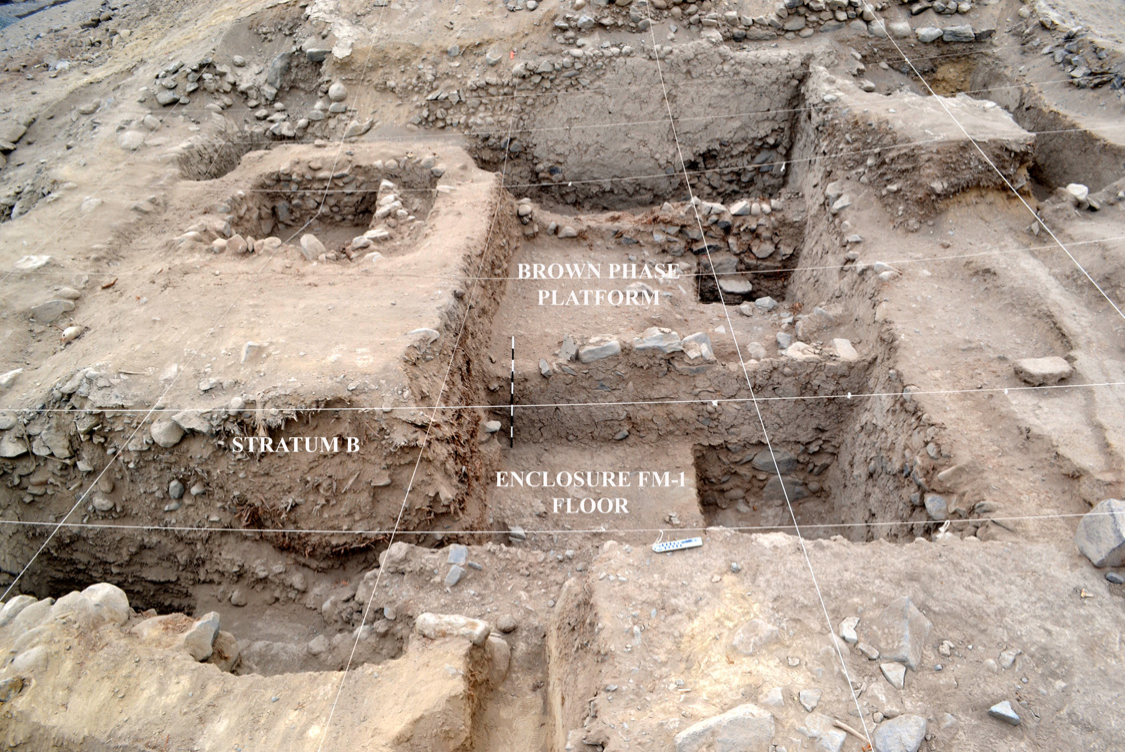
View from the west of Cerro del Gentil sunken patio. Note the Brown Phase platform and Enclosure FM-1.

**Fig 10:**
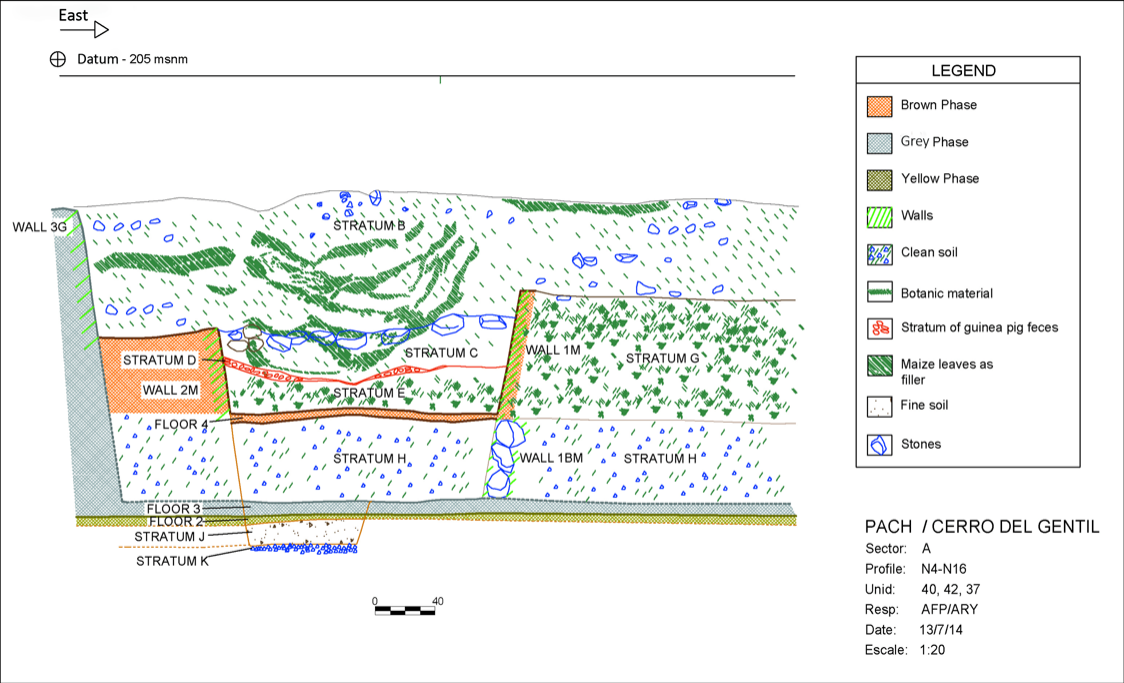
Profile West-East of sunken patio showing phases and strata.

The third phase, referred to as the “Brown” one, involved a substantial change in the size and use of the patio (Table 1 and Figs 9 and 10). It was at the end of this phase that the patio was abandoned with a series of elaborate rituals. This episode in the use of the patio dates from the mid 4^th^ to the mid 3^rd^ century BCE (Table 1 and Fig 8).

**Fig 11:**
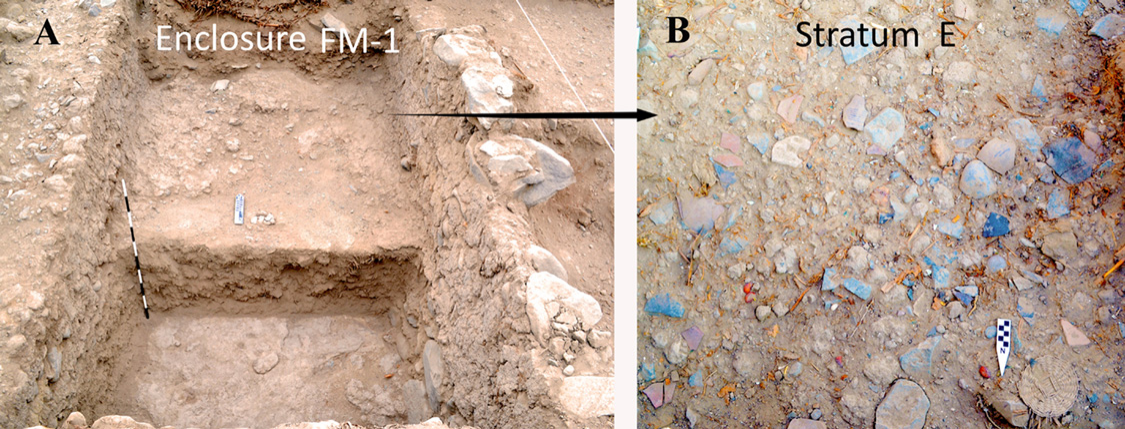
(A) View of Enclosure FM-1, (B) Detail of Stratum E.

Later, the architects first deposited a level of silty soil as a fill over the sunken patio of the Gray phase. This fill contained Paracas material. The fine silty texture indicates that liquid was used in this fill episode. The builders then constructed a wall (Wall 1M) that was filled in (Strata H and G). Stratum G was a clean fill while H was a silty clay loam mixed with objects. Over these strata we define the Brown Phase. The Brown phase platform is cardinally oriented and measures 7 m. north-south and 4 m. east-west. It was as high as the walls of the Gray phase court. The creation of a platform in the eastern side left a sunken rectangular space in the western sector of the old patio that measured 2 m. on the north-south axis and 7 m. east-west. This new area was 0.90 m deep. We called this space Enclosure FM-1 (Fig 9). Therefore, it represents a new use of the space marked principally by the intentional distortion of the original square sunken court (Figs 9 and 11A). This phase was used briefly, possibly related to the preparation of the space for its ritualized abandonment.

Finally, after this latest modification (Brown phase), the entire architectural space associated with this sunken patio was covered with soil in a series of fill events (Fig 10).

The beginning of what we describe as a ritual burial of the Brown phase patio, therefore, occurred with an archaeological level that we call Stratum E. This level was deposited immediately over the floor of Enclosure FM-1 and is between 30 and 40 centimeters thick with a maximum volume of 5.44 m^3^ (Figs 10 and 11). The matrix of this context is a silty mixed soil with large and medium sized rocks. This fill context is also characterized by the presence of a great density of cultural material, most notably ceramic, botanical, zoological, shell, and lithic artifacts (Fig 11B).

### Characteristics of Stratum E

Stratum E represents a great event that formally sealed the patio. Subsequent events (Strata C and B) represent post-abandonment activities during the Paracas period in which this patio did not function. Therefore, the composition of Stratum E is quite significant. Unlike the earlier ones, Stratum E has a very heterogeneous matrix with pottery, botanical and other objects. The following section describes the materials in Stratum E.

#### Ceramics

**Fig 12:**
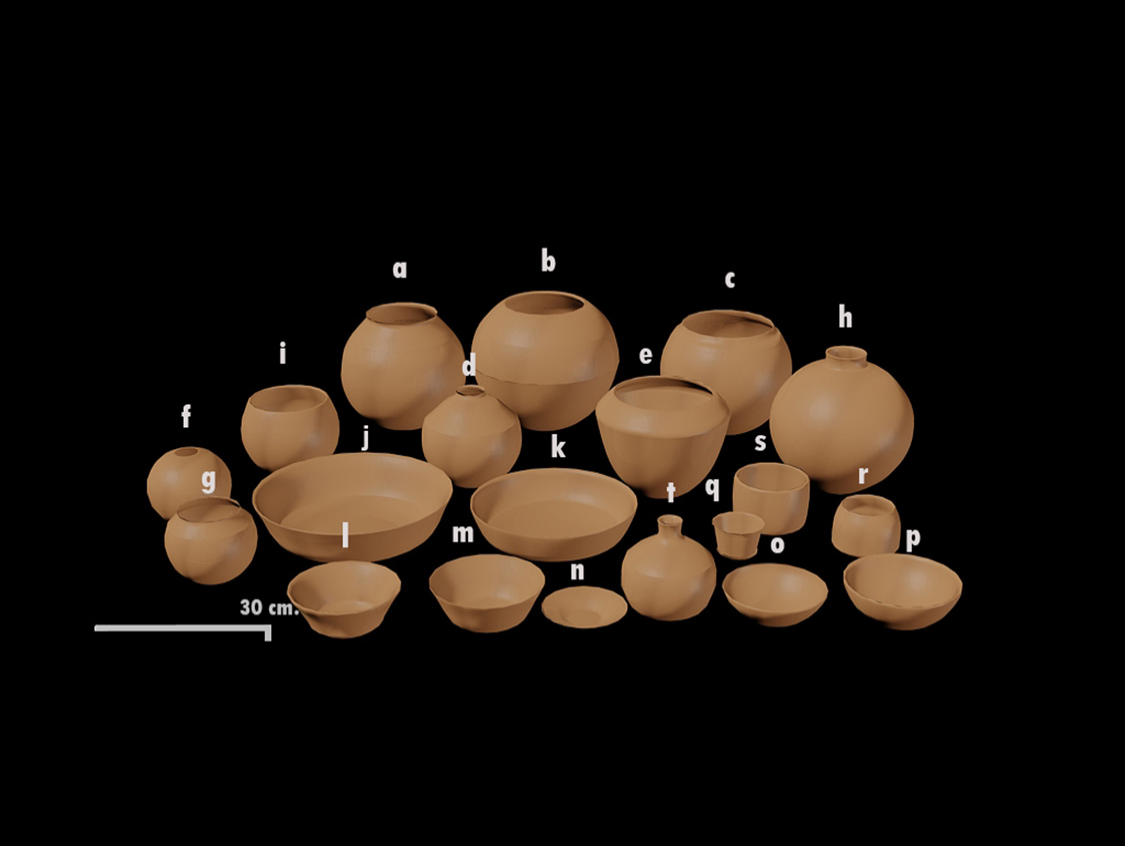
Digital reconstruction of the main pottery shapes found in Stratum E. (a) Neckless olla, rim with brace A; (b) convex neckless olla; (c) neckless olla, rim with brace B; (d) small neckless olla with angular shoulder; (e) neckless olla with angular shoulder; (f) convex neckless olla; (g) curved “S” rim, neckless olla; (h) necked jar; (i) hemispherical deep bowl; (j) oversized *tazón* with slightly vertical wall; (k) oversized hemispherical shallow bowl; (l) *tazón* with slightly concave wall; (m) *tazón with* slightly vertical wall; (n) plate with slightly vertical wall; (o) hemispherical shallow bowl; (p) Hemispherical slightly deep bowl; (q) small *tazón* with slightly concave wall; (r) convex glass; (s) small *tazón* with right angle wall.

We found 513 diagnostic pottery fragments in Stratum E virtually all of which were Paracas in style. Of these, 501 were vessel fragments, 8 were tools, and 4 were figurine fragments. We identified 9 large groups of vessels including ollas, necked jars, hemispherical bowls, hemispherical shallow bowls, plates, tazones, oversize bowls, glasses, and bottles (Fig 12 and S1 Table). Within these groups, we identified variants of these forms (S2 Table).

#### Botanical materials

Out of the more than 5500 recovered items, we have identified 15 species of plants. There is a wide variety of edible plants including maize, beans, pacae (*Inga feuillei*), yuca (*Manihot esculenta*), guayaba (*Psidium guajava*) and exotic fruits like palillo (*Campomanesia lineatifolia*) (S3 Table). We also found a number of non-comestibles. About a third of the sample was cotton (*Gossypium barbadense*) obviously used for textiles and rope. We also found junco (*Schoenoplectus sp*.) and caña brava (*Gynerium sagittatum*) that is used in the production of objects such as baskets, rope and litters. The presence of *Lagenaria siceraria* or bottle gourds is significant in that we found a variety of pyro-engraved ones.

#### Faunal remains

We identified a total of seven animal species out of a sample of 182 items (S4 Table). The most common species are Mountain parakeet (*Psilopsiagon aurifrons*), rodents (*muridae*), dogs (*canis lupus*), and camelids. The Mountain parakeet is common in the Chincha valley coastal region. Unlike other species, the dogs were found largely intact and without cut marks or other indicators of food preparation. These appear to be offerings and not foodstuffs. The rodents are most likely intrusive after the archaeological event. The camelids, in contrast were fragmented and had many cut marks. These were most certainly the remains of foodstuffs as the cut marks show signs of defleshing. There were also indications of post-mortem fractures indicating that meat may have been prepared for transport, there was post-depositional fracturing on a floor, or some other result of feasting or animal butchering. The single sample of an exotic bird (*Psiolpsiagon amazona* sp*)* was most likely an offering. We found not only the well-preserved bone of a mature bird but also part of the plumage. This species is found only in the eastern slopes of Andean South America.

#### Shellfish

We identified 20 shellfish species out of a total of almost 1430 items (S5 Table). It is notable that all of the malacological species were foodstuffs. The majority of the identified species at Cerro de Gentil are marine, with the exception of some small fresh water univalves. On the other hand, the most common marine shell 67% (962 items) is *Semimytilus algosus* or “chorito negro”. This could reflect a special kind of use, perhaps in feasts or festivals at Cerro del Gentil, because the site is 17 linear kilometers from the beach.

### Lithic artifacts

We found 69 lithic artifacts of which most (58) were tools. Tool types included granite and andesite *manos*, basalt and andesite knives, polishers, and spindle whorls.

### Special artifacts

**Fig 13:**
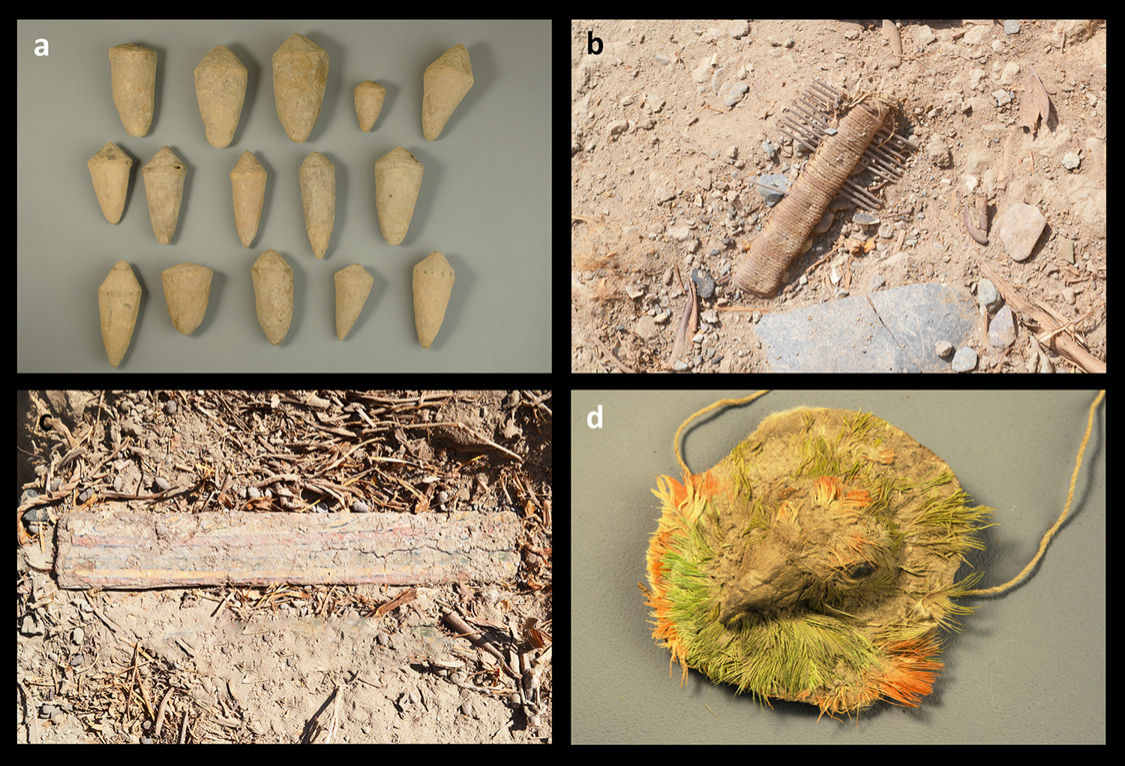
Rare objects discovered in the sunken patio in Stratum E. (a) Wooden cones: 5–10 cm x 3–4 cm; (b) Comb: ca. 5 x 9 cm; (c) Decorated wooden plaque: 30 cm x 5.5 cm.; (d) bird head medallion: 5 cm in diameter

**Fig 14:**
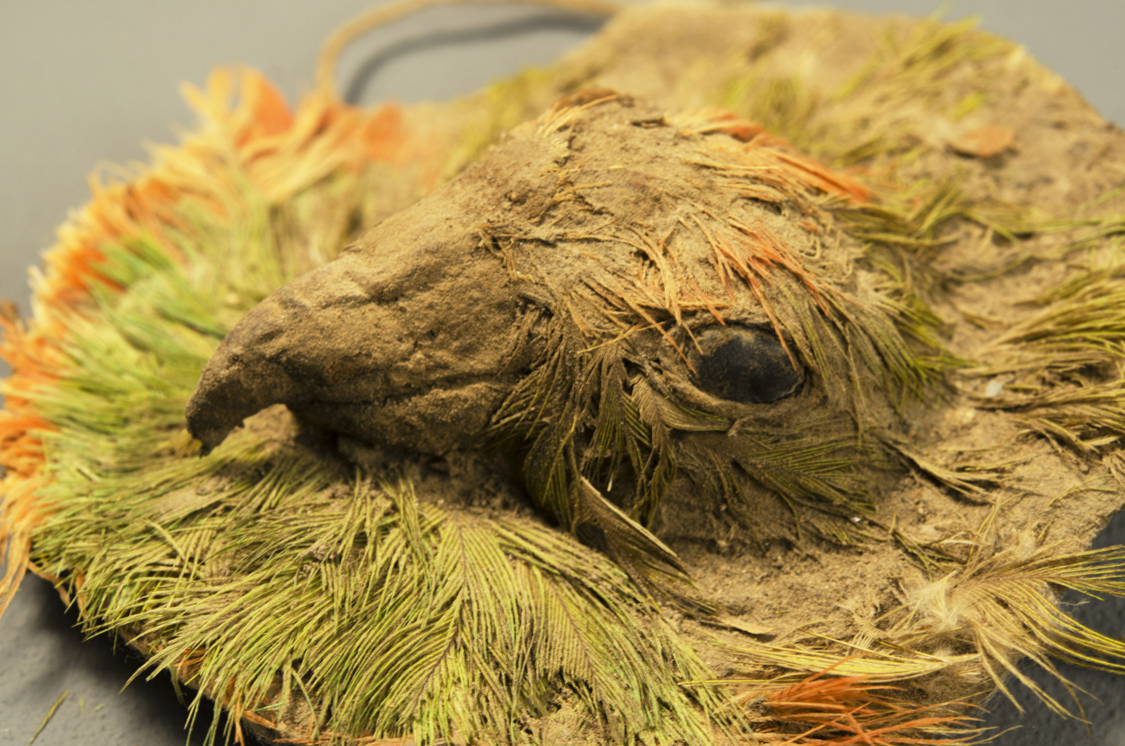
Detail of the bird head medallion.

We found a number of rare objects (Fig 13). These include some interesting wooden cones or “bottle caps” that were found in a cache (Fig 13A). We also found a “comb” (actually a tool for textile production) (Fig 11B), a pair of decorated wooden plaques (Fig 13C) and a “bird head medallion” (Fig 13D). This last one is a very impressive object and was made of unfired clay and molded into a bird head with feathers (Fig 14). The eyes of the bird were made with seeds, probably beans. The bird feathers belong to the specie *Aratinga wagleri* or red-fronted parakeet, a bird that is endemic to the Tumbes region in what is now the far north of Peru.

#### Post Stratum E depositions

After Stratum E was laid the patio was no longer used. Above this now-abandoned and covered patio we find about one meter of deposition in which we defined at least 8 distinct events (Strata C and B) (Fig 10). These last two levels were a rich mix of soil and artifacts, including many diagnostic Paracas ones. It is very clear archaeologically that the platform mound itself was not abandoned even though the patio was filled in and not in use. We do not know if this deposition (Strata C and B) occurred rapidly in a single, large event or if this filling took place over many separate ones.

## Results and Discussion

### Explaining the burial context of the sunken patio of Cerro del Gentil

The uniqueness of this context as represented by Stratum E is obvious and demands explanation. What type of social behavior created this archaeological signature? How can we explain the high density of common foodstuffs along with the offering of many high valued objects? In short, we propose that this event of Stratum E represents the remains of a great feast as described in the anthropological literature [23]. This feast was the central activity in a Paracas termination ceremony that lasted for a period of time, perhaps weeks if not months.

The pottery fragments stylistically correspond to vessels of Paracas Cavernas [15, 24] and phases Ocucaje 8 and 9 of the Ica Master sequence [25] (Fig 4). Many of these fragments together form individual vessels and show signs of domestic use. The simplest explanation is that the vessels were broken intentionally after use and were deposited with the other offerings, a practice seen in other archaeological examples [26–28]. The large quantity of ceramic vessels indicates a large consumption of food and drink. As we have mentioned before, these types of vessels are part of a complex of serving wares characterized by rims shapes that facilitate the pouring of liquids [29]. This feature is important, not only because it permits the contents to be placed inside and emptied easily but also because it allows people to readily see them [28, 29].

The neckless ollas could have been used to cook as well as to store foodstuffs. In this sense, it is important to note that we have identified olla fragments with evidence of having been placed in fires as well as those without such evidence (S1 Fig). Importantly, fine ollas with post-fire decoration do not have evidence of being used in hearths while the undecorated ones did have such evidence.

The production and consumption of alcoholic beverages is often central to feasts and the substantial presence of maize in our excavations supports this feasting model (and see [30–32]). These data suggest to us in effect that some of the ollas identified were used for the cooking of foodstuffs [29] while others were used to store and ferment *chicha*, the ubiquitous corn beer that is so central to Andean social life.

The pitchers were the vessels that were used to store and hold liquids. The advantage of small mouth diameter vessels obviously is that liquids do not spill. At the time in which the food and drink was consumed, it is likely that the liquid contents would have passed from the jars to the bottles and small *tazones*.

While it is true that in our case we did not find large amounts of liquid containers, we can say that they were comparatively higher in number than the other vessels, including small bowls. There are also large serving vessels with diameters between 26 and 50 cm that can contain comparatively more foodstuffs than vessels with more restricted necks. These vessels types are associated with feasting behaviors as defined by many ethnographers and archaeologists [26, 33, 34].

The large number of botanical remains found in the excavations supports this interpretation of a great feasting event. Most of the plants documented in the excavations are foodstuffs. One exception is the significant quantities of cotton (*Gossypium barbadense)*. Likewise the presence of rushes (junco or *Schoenoplectus sp*.*) and Gynerium sagittatum* (caña brava) are associated with the production of cloth, baskets and the like.

The faunal data are consistent with both feasting behaviors and use in domestic activities. This includes evidence of butchering meat and the presence of large quantities of shell [24, 35–37]. Instead, it is significant that fish bones are scarce in the deposits. This may simply be a case of cultural preferences or a question related to the preservation and transportation of fish from the coast located almost 15 km away from Cerro del Gentil.

Non domestic artifacts include combs, bottle stoppers, *atlatls*, litters, decorated wooden plaques, and the very rare clay bird face medallion decorated with feathers and a bean eye noted above. These are clearly non-domestic objects almost always interpreted to be part of a special ceremonial context when found archaeologically in such circumstances.

### A Termination Ritual at Cerro del Gentil

In sum, we can interpret this context as a grand event in which large quantities of food and drink were consumed, and in which both ordinary objects and highly valued ones were permanently deposited in the court area. This corresponds to what has been called a model of “political feasts” in the theoretical literature [33]. This kind of event has been amply identified in the Andes in similar archaeological contexts [26, 28, 38–43]. Many of the elements described in Cerro del Gentil have been found in these other sites.

We argue that this last feast preserved in Stratum E was a case of a “*Termination Ritual*” [44, 45]. This concept is defined as a ritual event that marks the final use of an important space. Termination rituals are commonly found in public architecture [45]. However, cases of such rituals are found in domestic contexts as well on a much smaller scale.

The concept of termination ritual is well developed in Mesoamerican archaeology. [46–49]. According to this work a termination ritual context should be characterized by: 1) the physical space where it occurred (a special construction, possibly associated with an elite or local leaders); 2) artifacts deposited in this space that are of high quality and rare and; 3) the overall context has evidence of the consumption of food and drink.

These kinds of contexts are not only found in Mesoamerica of course. There is substantial evidence for such rituals in the central Andes as well, and there are a wide variety of rituals expressed in different ways across space and time. They possibly appear in the Late Preceramic period (3000–1800 B.C.) where ritual burials were found on sites with “temples”, such as Kotosh [50, 51]. But it is the Formative (1800–200 B.C.), a period characterized by the surge of monumental architecture construction where we see substantial evidence for such behaviors. Here, we find buildings that were partially or totally covered with earth. Walls were destroyed and incorporated in these fill episodes and then offerings of various sorts were interred in these monuments.

A depositional context similar and temporally close to Cerro del Gentil, for instance, is that of Cerro Blanco in the Nepeña valley in the north coast [26]. Jorge Gamboa [45] has provided us an important analysis of termination ritual contexts for the Moche (250 BC-AD 800). Something similar occurred in architectural spaces at the famous Nazca site of Cahuachi in the south coast of Peru [52]. In this sense, different offering contexts such as the famous area at Conchopata [27] during the Wari state could be considered a kind of termination ritual as well.

These rituals occur in smaller scales as well. In Moquegua, for instance, Stanish [53] discovered a “killed” Tiwanaku kero placed inverted on a floor surface at the last use of a domestic structure in a very small Tiwanaku (ca. AD 950) hamlet. In a later site that dates to the immediate post-Tiwanaku period in the same valley, there was a set of common artifacts (polished throwing stones and polished camelid bone) left on a domestic floor as well in a manner that had to be the last behavior prior to abandonment [53].

More common are more elaborate rituals in more complex architectural contexts. Here this practice represents the conversion of sacred sites (such as temples or palaces) into other kinds of monuments such as tombs, pilgrimage destinations, commemoration of significant events, the conclusion of calendrical cycles and so forth [44].

The features described for Cerro del Gentil (as a first layer deposited on the floor of the Brown Phase) represents the initiation of the decommissioning process of the sunken patio space for an alternative and final social use at the site. This would be the ritual closing of this sunken space conducted longer than a few days or even weeks, but one in which this special place was laid to rest, as it were, fully removed from social practice and not used ever again as a sunken patio.

The nature of the architecture and the offerings indicate that a wide range of individuals participated in this event. One of the most striking features is the extraordinarily high variability of the pottery and other artifacts that were deposited in this termination event. It is very significant that of the large quantity of decorated objects placed in the patio during this event, none are very similar to each other. This indicates that at the very least there were different artisans producing these goods or that the artisans felt obligated to vary the art substantially. A second possibility is that many objects were curated by the local population, objects that had been collected from different peoples over many years. A third possibility is that the highly individualized nature of the offerings indicates that people from a wide area or from different groups participated in the closing event bringing their own culturally- or geographically-specific objects. The existence of one entire parrot from the eastern slopes as well as highland camelid bone reinforces the proposition that people who participated in this event came from, or at least imported objects from, a wide area from the highlands to the coast. Also, it is possible that some of these exotic materials could have arrived to Cerro del Gentil via down-the-line trade through an existing Late Paracas period trade network

It is important to emphasize that there were no architectural alterations of the sunken patio after the Brown Phase. There were however, at least 8 distinct events (after the large termination one of Stratum E) that we defined that were part of this final use of the site. There were very thin but dense levels of silty soil similar that found in the first level. Again, the silty textures indicate the use of liquids in this ritual event. The carbon dates of these latter events are statistically identical to the final termination one (Table 1 and Fig 8). In other words, the termination event and the subsequent smaller events were conducted very close in time. Therefore, we can surmise that after the final large offering event we see a series of smaller ones, perhaps representing elaborate feasting episodes lasting over weeks or even months.

## Conclusion

The site of Cerro del Gentil is a satellite ceremonial mound of the larger and permanent Paracas occupation in the lower valley. The site served for several centuries as a place of economic exchange through fairs, accompanied by feasts and ceremonies of various kinds. At a certain point in history, the political and economic structure that maintained this complex regional system collapsed. The Paracas period patio on Cerro del Gentil was ritually abandoned with a massive termination ceremony. The subsequent use of the platform mound by the Topará polity including the building of a stone structure and the filling in of the patio area. Excavations in the Topará sector revealed that there was no patio. The tradition of sunken patio construction, a style that developed in the 3^rd^ millennium BCE in the north coast [54] and continued in the Paracas period, abruptly ended. We do not understand the complex factors involved in the collapse of the sunken court tradition, but it clearly is part of the process of the transition from Paracas to later polities; it is a fascinating subject for future research.

## Supporting Information

S1 Fig Graphic of evidence of carbonization on vessels. (TIF)

S1 Table Ceramic forms recovered in Stratum E. (DOCX)

S2 Table Ceramic variants recovered in Stratum E. (DOCX)

S3 Table Botanical remains recovered in Stratum E. (DOCX)

S4 Table Faunal remains recovered in Stratum E. (DOCX)

S5 Table Shellfish recovered in Stratum E. (DOCX)
