# Supplementary figures and images for: The Final Days of Paracas in Cerro del Gentil, Chincha Valley, Peru
Source: PLoS One. 2016 May 4;11(5):e0153465. doi: 10.1371/journal.pone.0153465 (PMC4856392; doi:10.1371/journal.pone.0153465)

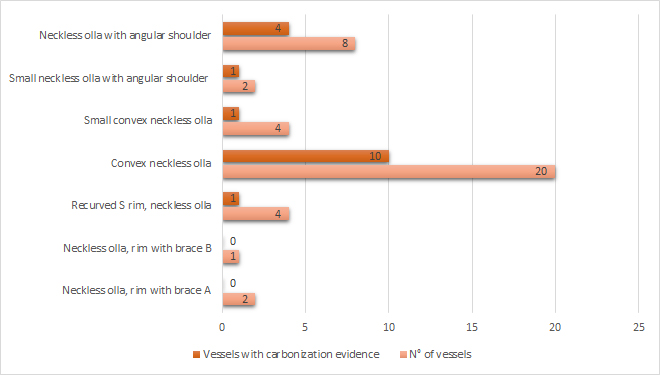

Supplement: S1 Fig — (TIF) [file pone.0153465.s001.tif]
