# Supplementary material for: The Final Days of Paracas in Cerro del Gentil, Chincha Valley, Peru
Source: PLoS One. 2016 May 4;11(5):e0153465. doi: 10.1371/journal.pone.0153465 (PMC4856392; doi:10.1371/journal.pone.0153465)
Supplement: S1 Table — (DOCX) [file pone.0153465.s002.docx]

| **General Shapes (3)** | **Specific Shapes (2)** | **Tipology (1)** | **Total (1)** | **%(1)** | **Total (2)** | **%(2)** | **Total (3)** | **%(3)** |
| --- | --- | --- | --- | --- | --- | --- | --- | --- |
| Unrestricted Shapes | Hemispherical bowl | Hemispherical slightly deep bowl | 43 | 8,38% | 43 | 8,38% | 156 | 30,41% |
|  | Hemispherical shallow bowl | Hemispherical shallow bowl | 50 | 9,75% | 53 | 10,33% |  |  |
|  |  | Oversized, Hemispherical shallow bowl | 3 | 0,58% |  |  |  |  |
|  | Tazones | Tazón with slightly concave walled | 19 | 3,70% | 57 | 11,11% |  |  |
|  |  | Tazón with slightly vertical walled | 27 | 5,26% |  |  |  |  |
|  |  | Oversized tazón with slightly vertical walled | 4 | 0,78% |  |  |  |  |
|  |  | Small tazón with right angle walled | 6 | 1,17% |  |  |  |  |
|  |  | Small tazón with slightly concave walled | 1 | 0,19% |  |  |  |  |
|  | Plates | Plate with slightly vertical walled | 3 | 0,58% | 3 | 0,58% |  |  |
| Restricted Shape | Ollas | Neckless olla, rim with brace A | 2 | 0,39% | 41 | 7,99% | 63 | 12,28% |
|  |  | Neckless olla, rim with brace B | 1 | 0,19% |  |  |  |  |
|  |  | Recurved S rim, neckless olla | 4 | 0,78% |  |  |  |  |
|  |  | Convex neckless olla | 20 | 3,90% |  |  |  |  |
|  |  | Neckless olla with angular shoulder | 8 | 1,56% |  |  |  |  |
|  |  | Small convex neckless olla | 4 | 0,78% |  |  |  |  |
|  |  | Small neckless olla with angular shoulder | 2 | 0,39% |  |  |  |  |
|  | Glasses | Convex glass | 1 | 0,19% | 1 | 0,19% |  |  |
|  | Hemispherical bowl | Hemispherical deep bowl | 8 | 1,56% | 8 | 1,56% |  |  |
|  | Bottles | Spouted bottle | 4 | 0,78% | 4 | 0,78% |  |  |
|  | Necked jar | Necked jar | 7 | 1,36% | 7 | 1,36% |  |  |
| Tools | Tools | Potter's wheel | 2 | 0,39% | 2 | 0,39% | 8 | 1,56% |
|  |  | Spindle whorls | 6 | 1,17% | 6 | 1,17% |  |  |
| Others | Modeling | Figurine | 4 | 0,78% | 4 | 0,78% | 4 | 0,78% |
| Indeterminados (diagnósticos) | Indeterminate | Indeterminate | 284 | 55,36% | 284 | 55,36% | 284 | 55,36% |
| **TOTAL** | | | **513** | 100,00% | **513** | **100,00%** | **513** | **100,00%** |
