# Supplementary material for: The Final Days of Paracas in Cerro del Gentil, Chincha Valley, Peru
Source: PLoS One. 2016 May 4;11(5):e0153465. doi: 10.1371/journal.pone.0153465 (PMC4856392; doi:10.1371/journal.pone.0153465)
Supplement: S2 Table — (DOCX) [file pone.0153465.s003.docx]

| **Function (3)** | **Specific Shapes (2)** | **Tipology (1)** | **Total (1)** | **%(1)** | **Total (2)** | **%(2)** | **Total (3)** | **%(3)** |
| --- | --- | --- | --- | --- | --- | --- | --- | --- |
| Vessel for food production | Ollas | Neckless olla, rim with brace A | 2 | 0,92% | 41 | 18,89% | 48 | 22,12% |
|  |  | Neckless olla, rim with brace B | 1 | 0,46% |  |  |  |  |
|  |  | Recurved S rim, neckless olla | 4 | 1,84% |  |  |  |  |
|  |  | Convex neckless olla | 20 | 9,22% |  |  |  |  |
|  |  | Neckless olla with angular shoulder | 8 | 3,69% |  |  |  |  |
|  |  | Small convex neckless olla | 4 | 1,84% |  |  |  |  |
|  |  | Small neckless olla with angular shoulder | 2 | 0,92% |  |  |  |  |
|  | Necked jar | Necked jar | 7 | 3,23% | 7 | 3,23% |  |  |
| Food service vessel | Hemispherical bowl | Hemispherical slightly deep bowl | 43 | 19,82% | 51 | 23,50% | 169 | 77,88% |
|  |  | Hemispherical deep bowl | 8 | 3,69% |  |  |  |  |
|  | Hemispherical shallow bowl | Hemispherical shallow bowl | 50 | 23,04% | 50 | 23,04% |  |  |
|  | Plates | Plate with slightly vertical walled | 3 | 1,38% | 3 | 1,38% |  |  |
|  | Tazones | Tazón with slightly concave walled | 19 | 8,76% | 46 | 21,20% |  |  |
|  |  | Tazón with slightly vertical walled | 27 | 12,44% |  |  |  |  |
|  | Oversize bowl | Oversized, Hemispherical shallow bowl | 3 | 1,38% | 7 | 3,23% |  |  |
|  |  | Oversized tazón with slightly vertical walled | 4 | 1,84% |  |  |  |  |
|  | Glasses | Small right angle walled bowl | 6 | 2,76% | 8 | 3,69% |  |  |
|  |  | Small slightly concave walled bowl | 1 | 0,46% |  |  |  |  |
|  |  | Convex glass | 1 | 0,46% |  |  |  |  |
|  | Bottle | Spouted bottle | 4 | 1,84% | 4 | 1,84% |  |  |
| **TOTAL** | | | **217** | 100,00% | **217** | 100,00% | **217** | **100,00%** |
