# Supplementary material for: The Final Days of Paracas in Cerro del Gentil, Chincha Valley, Peru
Source: PLoS One. 2016 May 4;11(5):e0153465. doi: 10.1371/journal.pone.0153465 (PMC4856392; doi:10.1371/journal.pone.0153465)
Supplement: S3 Table — (DOCX) [file pone.0153465.s004.docx]

S6. Botanical remains recovered in Stratum E

| **Class** | **Family** | **Species** | **Total** | **%** |
| --- | --- | --- | --- | --- |
| Monocotolydoneae | Myrtaceae | *Psidium guajava* | 76 | 1,36% |
|  |  | *Campomanesia lineatifolia* | 485 | 8,71% |
|  | Cucurbitaceae | *Lagenaria siceraria* | 112 | 2,01% |
|  | Bromileaceae | *Tillandsia sp.* | 25 | 0,45% |
|  | Poaceae | *Zea mays* | 743 | 13,34% |
|  |  | *Zea mays L.* | 326 | 5,85% |
|  |  | *Gynerium sagittatum* | 14 | 0,25% |
|  | Cyperaceae | *Schoenoplectus sp.* | 52 | 0,93% |
| Dicotyledoneae | Malvaceae | *Gossypium barbadense* | 1699 | 30,50% |
|  | Fabaceae | *Phaseolus vulgaris* | 87 | 1,56% |
|  |  | *Phaseolus lunatus* | 553 | 9,93% |
|  |  | *Canavalia plagiosperma* | 949 | 17,03% |
|  |  | *Inga feuillei* | 334 | 6,00% |
|  |  | *Arachis hypogaea* | 364 | 6,53% |
|  | Euphorbiaceae | *Manihot esculenta* | 48 | 0,86% |
| Indeterminate | Indeterminate | Indeterminate | 6 | 0,11% |
| **TOTAL** |  |  | 5571 | 100,00% |
