# Supplementary material for: The Final Days of Paracas in Cerro del Gentil, Chincha Valley, Peru
Source: PLoS One. 2016 May 4;11(5):e0153465. doi: 10.1371/journal.pone.0153465 (PMC4856392; doi:10.1371/journal.pone.0153465)
Supplement: S4 Table — (DOCX) [file pone.0153465.s005.docx]

| **Filo** | **Class** | **Family** | **Species** | **Total** | **%** |
| --- | --- | --- | --- | --- | --- |
|  |  |  |  |  |  |
| Mollusca | Bivalvia | Pectinidae | *Argopecten purpuratus* | 1 | 0,07% |
|  |  | Mytilidae | *Aulacomya atra* | 95 | 6,65% |
|  |  |  | *Brachidontes sp.* | 1 | 0,07% |
|  |  |  | *Brachidontes variabilis* | 53 | 3,71% |
|  |  |  | *Choromytilus chorus* | 11 | 0,77% |
|  |  |  | *Semimytilus algosus* | 962 | 67,32% |
|  |  | Donaciae | *Donax obesulus* | 20 | 1,40% |
|  |  | Fasciolariidae | *Filifusus Filamentosus* | 6 | 0,42% |
|  |  | Mactridae | *Mulinia edulis* | 62 | 4,34% |
|  |  |  | *Mulinia sp.* | 4 | 0,28% |
|  | Gastropoda | Calyptraeidae | *Calyptraea trochiformis* | 12 | 0,84% |
|  |  |  | *Crepipatella lingulata* | 17 | 1,19% |
|  |  | Fissurellidae | *Fissurella sp.* | 7 | 0,49% |
|  |  | Turbinidae | *Prisogaster sp.* | 1 | 0,07% |
|  |  | Bulimidae | *Scutalus sp.* | 2 | 0,14% |
|  |  | Tegulidae | *Tegula atra* | 118 | 8,26% |
|  |  |  | *Tegula corteziana* | 2 | 0,14% |
|  |  |  | *Tegula tridentata* | 10 | 0,70% |
|  |  | Muricidae | *Thais sp.* | 18 | 1,26% |
| Arthropoda | Maxillopoda | Balanidae | *Perforatus perforatus* | 20 | 1,40% |
|  |  | Crustáceo N/I | Crustáceo N/I | 5 | 0,35% |
| Indeterminado | Indeterminado | Indeterminado | Indeterminado | 2 | 0,14% |
| **Total** | | | | 1429 | 100,00% |
